# Production and purification of a soluble hydrogenase from *Ralstonia eutropha* H16 for potential hydrogen fuel cell applications

**DOI:** 10.1016/j.mex.2016.03.005

**Published:** 2016-03-22

**Authors:** Bat-Erdene Jugder, Helene Lebhar, Kondo-Francois Aguey-Zinsou, Christopher P. Marquis

**Affiliations:** a School of Biotechnology and Biomolecular Sciences, University of New South Wales, Sydney 2052 Australia; b MERLin group, School of Chemical Engineering, University of New South Wales, Sydney 2052 Australia

**Keywords:** Soluble hydrogenase purification from *Ralstonia eutropha*, Soluble hydrogenase, *Ralstonia eutropha*, *Cupriavidus necator*, Purification, Hydrogen oxidation

## Abstract

The soluble hydrogenase (SH) from *Ralstonia eutropha* H16 is a promising candidate enzyme for H_2_-based biofuel application as it favours H_2_ oxidation and is relatively oxygen-tolerant. In this report, bioprocess development studies undertaken to produce and purify an active SH are described, based on the methods previously reported [1], [2], [3], [4]. Our modifications are:

• Upstream method optimizations were undertaken on heterotrophic growth media and cell lysis involving ultrasonication.
• Two anion exchangers (Q Sepharose and RESOURCE Q) and size exclusion chromatographic (Superdex 200) matrices were successfully employed for purification of a hexameric SH from *R. eutropha*.
• The H_2_ oxidizing activity of the SH was demonstrated spectrophotometrically in solution and also immobilized on an EPG electrode using cyclic voltammetry.

## Graphical abstract

**Figure:**
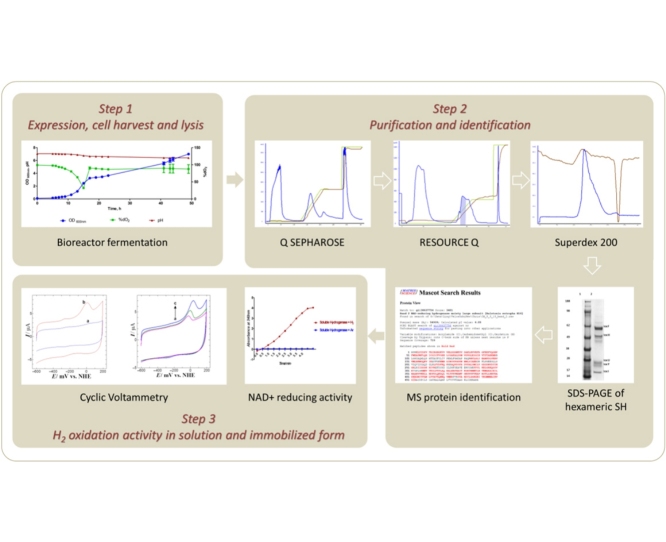

## Method details

### Step 1: soluble hydrogenase expression, cell harvest and lysis

*Ralstonia eutropha* H16 is a strictly aerobic, facultatively chemolithoautotrophic bacterium. It is able to grow in both autotrophic and heterotrophic conditions [5]. Autotrophic growth requires a complicated gas mixture of H_2_, O_2_ and CO_2_ at a ratio of 8:1:1 v/v/v. Heterotrophic growth is supported by a variety of organic substrates, but fructose and glycerol are widely used with this bacterial species, as the latter has been proven to provide catabolic derepression for hydrogenase expression [1]. Nevertheless, the fact that different compositions of FGN (Fructose-Glycerol-Nitrogen) media had been reported previously [1], [2], [3], [4] determined further investigation was necessary to find an optimal composition of FGN medium for this study. Several modifications of FGN medium (e.g., changing carbon source concentration, supplementing with trace element solutions) were investigated to achieve the highest cell growth and SH activity. An optimal cell lysis protocol for *R. eutropha* was also developed.

## Materials

• *R. eutropha* H16 (*Cupriavidus necator*, DSM 428)
• Minimal medium FGN consisting of 100 mL 10X H16 buffer, 850 mL Milli-Q water, 10 mL 20% NH_4_Cl, 1 mL 20% MgSO_4_ × 7H_2_O, 1 mL 1% CaCl_2_ × 2H_2_O, 1 mL 0.5% FeCl_3_ × 6H_2_O, 1 mL 0.02% NiCl_2_, 5 mL 40% fructose and 5 mL 40% glycerol. 10X H16 buffer contains 90 g Na_2_HPO_4_ × 12H_2_O and 15 g KH_2_PO_4_ topped up to 1 L Milli-Q water (pH 7.0). SL-6 trace elements solution [1]: 100 mg ZnSO_4_ × 7H_2_O, 30 mg MnCl_2_ × 4H_2_O, 300 mg H_3_BO_3_, 200 mg CoCl_2_ × 6H_2_O, 15 mg CuSO_4_ × 5H_2_O, 20 mg NiCl_2_ × 6H_2_O and 30 mg Na_2_MoO_4_ × 2H_2_O dissolved in 1 L Milli-Q water. This was sterilized via 0.2 μm filtration. Among the different compositions of FGN media analyzed, it was observed that the cells grown in the FGN medium supplemented with 0.2% fructose and 0.2% glycerol without any trace elements solution (SL-6) but containing additional NiCl_2_ exhibited good growth and SH activity.
• 50 mM KPi (potassium phosphate) buffer: 21.1 mL 1 M KH_2_PO_4_, 28.9 mL 1 M K_2_HPO_4_ and Milli-Q water up to 1 L (pH 7.0).
• cOmplete, EDTA-free Protease inhibitor (Roche Applied Science, Germany)
• DNase I (Sigma, Australia)
• 1 M NaOH for pH adjustment
• Laboratory scale glass bioreactor (Applikon, The Netherlands) with a working volume of 5 L
• Biowave Cell Density Meter (Biochrom, England) to measure optical density at 600 nm (OD_600nm_)
• Centrifuge with capacity to spin at 20,000 *g* at 4 °C
• Branson Digital Sonifier equipped with 1/8 Tapered Microtip (Branson Ultrasonics Corporation, USA)

## Procedure

1. Preparation of the bioreactor inoculum was initiated by picking a single colony of *R. eutropha* H16 from a FGN agar plate and using this to inoculate 5 mL FGN media in a 15 mL centrifuge (Falcon) tube. Cells were incubated at 30 °C on a shaker overnight. The overnight inoculum was transferred to a 1 L sterile baffled shake flask containing 100 mL FGN media. The 100 mL pre-culture was grown overnight to serve as the inoculum for the bioreactor fermentation. The batch fermentation was undertaken in a laboratory scale glass bioreactor with a working volume of 5 L. Growth was monitored by measuring the OD_600nm_ of the fermentation broth.
2. The bioreactor was operated at 30 °C with an agitation speed of 300–350 rpm and an air flow rate of 1–2 L/min. The initial pH of the culture was 7.0–7.1 and pH was not allowed to drop below 6.4 via automatic addition of 1 M NaOH when the pH reached this setpoint. For obtaining the highest SH activity, FeCl_3_ and NiCl_2_ were added at 15 h to a final concentration of 10 μM and 1 μM, respectively.
3. After 48 h, cells were harvested by centrifugation (10,000 *g* at 4 °C for 10 min) followed by washing in an appropriate volume of 50 mM KPi buffer (pH 7.0). The cell pellets were stored at −80 °C.
4. The cell pellet was thawed and resuspended in 50 mM KPi buffer containing cOmplete, EDTA-free Protease inhibitor and DNase I (final concentration 20 μg/ml). A ratio of cell wet weight and the resuspension buffer of 1:5 was used. The cell suspension was sonicated using a Branson Digital Sonifier equipped with 1/8 Tapered Microtip by applying 50% amplitude. Various duty cycles for finding the optimal cell disruption condition were investigated. Cell debris was removed by centrifugation at 20,000 *g* for 50 min at 4 °C.

### Step 2*:* soluble hydrogenase purification and identification

The purification process described here for SH was designed using modern chromatography matrices not available when the original methods were described. The performance of each of the previously employed matrices (DEAE Sepharose, Phenyl Sepharose and Superdex 200) [3], [6], [7] and matrices not previously described for this process (Q Sepharose and RESOURCE Q) were optimized with respect to resolution and recovery. For example, the performance of Phenyl HP was investigated applying conditions as previously published [6]: a two-step gradient of 200–50 mM KPi and 10–0 mM KPi was applied for four and three column volumes, respectively, and the enzyme was expected to be eluted during the latter gradient. However, the SH failed to bind to the column (data not shown). The newly developed downstream process involved a traditional ammonium sulphate precipitation followed by two consecutive ion exchange steps (employing Q Sepharose and RESOURCE Q) and a final size exclusion chromatography step (Superdex 200). The recruitment of the second anion exchanger RESOURCE Q column following the first anion exchanger Q Sepharose resulted in significantly improved enzyme purity. The newly developed bioprocess steps designed in this study resulted in an 18.7% yield and a 13.1 fold purification to ultimately obtain the pure active SH preparation. All purification steps were performed at 4 °C under aerobic conditions.

### Materials

• Saturated ammonium sulphate solution ((NH_4_)_2_SO_4_) for ammonium sulphate fractionation
• KPi 50 mM buffer with or without 1 mM EDTA (pH 7.0)
• Slide-A-Lyzer Dialysis cassettes 10 K MWCO (Thermo Scientific, USA) or SnakeSkin Dialysis Tubing, 10 K MWCO (Thermo Scientific, USA) for protein dialysis
• Unless otherwise stated, all chromatographic media and systems were obtained from GE Healthcare. All liquid chromatographic steps were performed at 4 °C on a calibrated ÄKTAexplorer™ system, controlled by UNICORN™ software. All chromatography buffers were filtered through 0.22 μm filter membranes (Millipore, USA) and degassed under vacuum. The protein samples were routinely filtered through 0.22 μm filter units (Millipore, USA) prior to loading sample on to the columns. The columns used were a pre-packed HiTrap Q Sepharose FF 1 mL column or a manually packed Q Sepharose FF 7.8 mL column, RESOURCE Q 1 mL column and Superdex 200 10/300 GL (analytical grade) column.
• Vivaspin^®^20 10 K MWCO centrifugal concentrator (GE Healthcare, Sweden)
• Protein concentrations were routinely determined according to the BCA (bicinchoninic acid) method using the Pierce^®^ BCA Protein Assay Kit (Thermo Fisher Scientific, USA) [8].
• SDS-PAGE was performed using NuPAGE^®^ Novex^®^ 4–12% Bis-Tris (Life Technologies, USA) pre-cast polyacrylamide gels. SeeBlue^®^ Plus2 Pre-Stained Standard (Life Technologies, USA) was used as protein standard. Protein gels were stained with either the GelCode Blue Stain Reagent (based on Coomassie dye G-250, (Thermo Fisher Scientific, USA)) or the Silver Stain Plus kit (Bio-Rad, Australia) according to the manufacturer’s instructions.
• Access to mass spectrometric analysis for protein identification

### Procedure

**Fig. 1:**
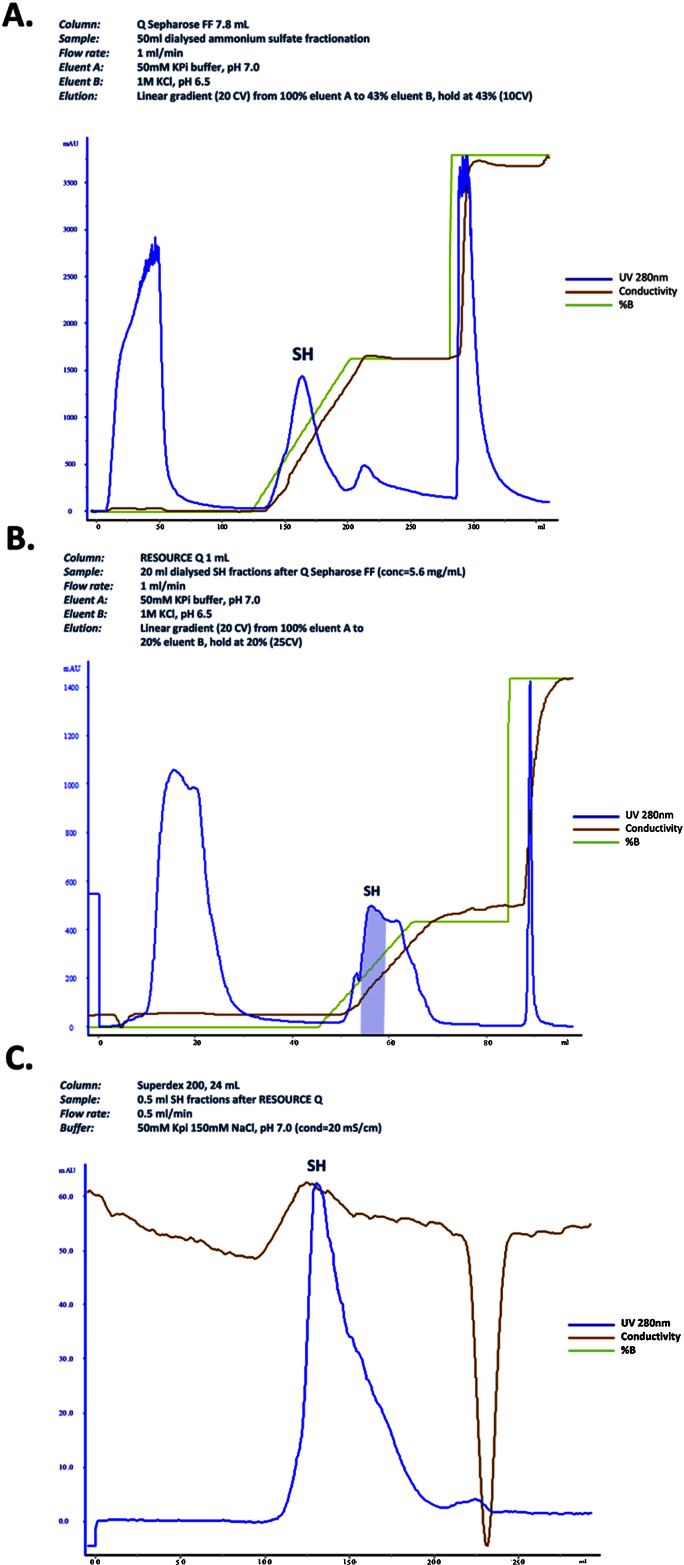
Purification of the soluble hydrogenase (SH). A) Q Sepharose FF chromatogram, stepwise gradients. SH active fractions were eluted in the first peak (Abs at 280 nm) during a linear gradient of 0–43% B. B) RESOURCE Q chromatogram, stepwise gradients. SH active fractions were eluted in the first peak (shadowed) during a linear gradient of 0–20% B. C1) Superdex 200 chromatogram. 50 mM KPi buffer supplemented with 150 mM NaCl.

**Fig. 2:**
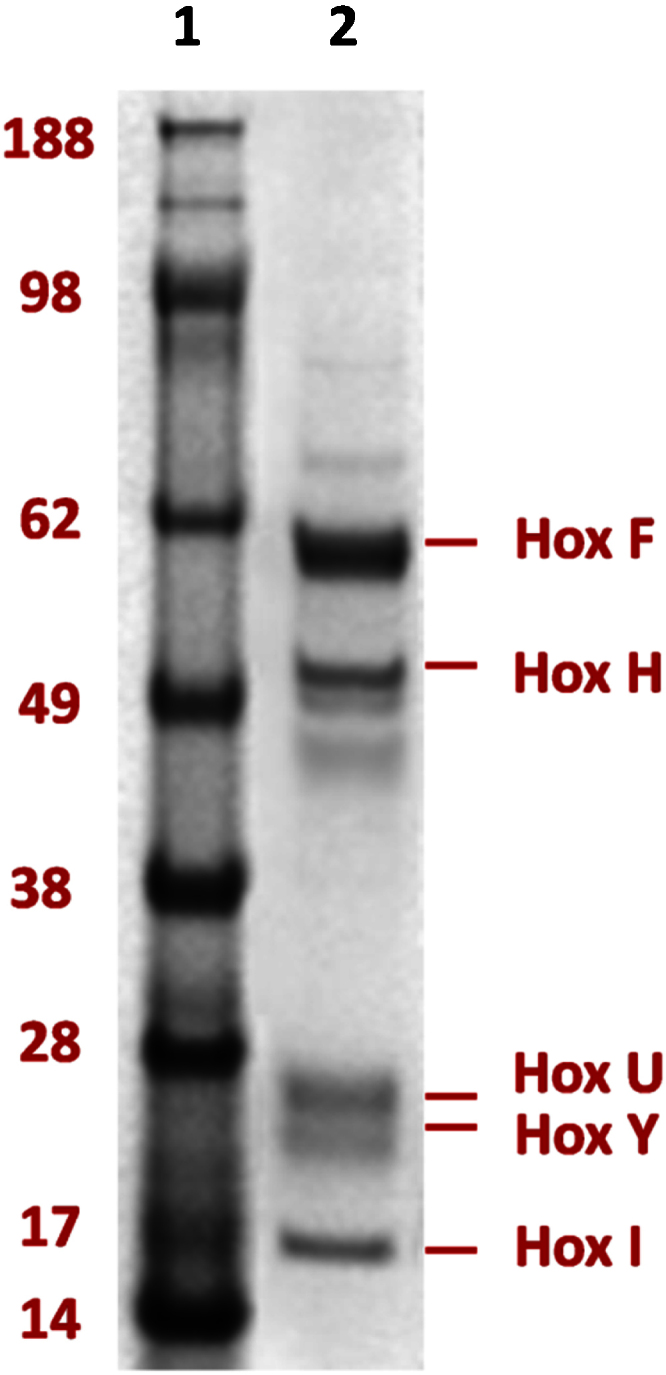
SDS-PAGE of the pure soluble hydrogenase. Lane 1: SeeBlue^®^ Plus2 Pre-Stained Standard; Lane 2: The SH fraction from the 50 mM KPi buffer with 150 mM NaCl gel filtration; 10 μg protein was loaded into the well. The hexameric SH comprises of 5 subunits; Hox I (two copies), HoxY, HoxU, HoxH, Hox F.

1. The ammonium sulphate fractionation was performed as described previously [6]. Briefly, the cell-free extract was fractionated by addition of saturated ammonium sulphate solution to 35% saturation. After incubation on ice and centrifugation at 10,000 *g*, the resulting supernatant was brought to 60% ammonium sulphate saturation. The precipitated enzyme was dissolved in 50 mM KPi buffer, pH 7.0.
2. The protein solution was dialysed twice for 3 h against 2 L of KPi 50 mM buffer containing 1 mM EDTA in order to remove the ammonium sulphate. Depending on the sample volume, either Slide-A-Lyzer Dialysis cassettes 10 K MWCO or SnakeSkin Dialysis Tubing, 10 K MWCO was used.
3. Initial anion exchange chromatography: A pre-packed HiTrap Q Sepharose FF 1 mL column was used for the first chromatography step. Eluent buffers used were 50 mM KPi (pH 7.0) and 1 M KCl (pH 6.5) as previously used for DEAE FF. Various linear and stepwise gradients (30%, 43%, and 100% of 1 M KCl) were applied to elute the active protein. For the process scale-up, a manually packed Q Sepharose FF 7.8 mL column was used with the same settings. Based on the calculations from the previous performance of Q Sepharose FF 1 mL columns, a linear gradient of 0–43%B (up to approximately 430 mM KCl) was applied for a complete recovery of SH (Fig. 1A). After a complete capture of all SH containing fractions in the first peak after elution start, two other peaks containing contaminants were eluted, and the activity assays for the fractions collected from each peak were undertaken. The active fractions were pooled, and dialyzed against 2 L 50 mM KPi buffer (pH 7.0) containing 1 mM EDTA at 4 °C.
4. Second anion exchange chromatography: For further purification of the protein, the dialyzed protein solution exhibiting SH activity eluted from Q Sepharose FF was loaded on to a RESOURCE Q 1 mL column pre-packed with SOURCE™ 15Q medium. This resin is comprised of smaller monodispersed beads (∼15 μm) compared to those in Q Sepharose FF (45–165 μm), which generally results in improved resolution. The eluent buffers, the flow rate and fraction sizes were equivalent to those used in the Q Sepharose FF anion exchange columns. Based on the results from the previous linear gradient run, the elution gradient was narrowed to 20%B (0–200 mM KCl). During a linear gradient of 0–20%B, the second peak spike of the first peak contains the highest SH activity (Fig. 1B).
5. Size exclusion chromatography: The combined active fractions generated after the second anion exchange chromatography column step, RESOURCE Q, were concentrated by membrane ultrafiltration in a Vivaspin^®^20 10 K MWCO centrifugal concentrator to a final volume of approximately 0.5 mL. The protein solution was then loaded on to a Superdex 200 10/300 GL (analytical grade) column using a 500 μL sample loop. Having considered that the recommended ionic strength of the buffer is ≥20 mS/cm according to the manufacturer’s instruction, the performance of the gel filtration with a higher ionic strength buffer, 50 mM KPi containing 150 mM NaCl (with 20 mS/cm conductivity), compared to previously used 50 mM KPi only buffer (with 7 mS/cm conductivity) [3], [6] was evaluated to avoid non-specific binding. Fractions were manually collected at a flow rate of 0.5 mL/min. The SH active fractions were combined and re-concentrated (Fig. 1C). Prior to storing at −80 °C, the SH solution was headspace flushed with nitrogen.
6. Mass spectrometric analysis was carried out at the Bioanalytical Mass Spectrometry Facility, University of New South Wales, Australia. The five protein bands that represent the expected six subunits of the hexameric SH after Superdex 200 were excised from the Commassie Blue stained gel shown in Fig. 2 and subjected to mass spectrometry analysis. Briefly, trypsin-digested peptides were separated by nano-LC using an Ultimate 3000HPLC and autosampler system (Dionex, Netherlands). Samples (2.5 μL) were concentrated and desalted onto a micro C18 precolumn (500 μm × 2 mm, Michrom Bioresources, USA) with H_2_O:CH_3_CN (98:2, 0.05% TFA) at 15 μL/min. After a 4 min wash the pre-column was switched (Valco 10 port valve, Dionex) into line with a fritless nano column (75 μ × ∼10 cm) containing C18 media (5 μ, 200 Å Magic, Michrom) manufactured according to Gatlin et al. [9]. Peptides were eluted using a linear gradient of H_2_O:CH_3_CN (98:2, 0.1% formic acid) to H_2_O:CH_3_CN (64:36, 0.1% formic acid) at 250 nL/min over 30 min. High voltage (2000 V) was applied to low volume tee (Upchurch Scientific) and the column tip positioned ∼0.5 cm from the heated capillary (T = 280 °C) of an Orbitrap Velos (Thermo Electron, Germany) mass spectrometer. Positive ions were generated by electrospray and the Orbitrap operated in data dependent acquisition mode (DDA). Peak lists were generated using Mascot Daemon/extract_msn (Matrix Science, England) using the default parameters, and submitted to the database search program Mascot (version 2.2, Matrix Science). Search parameters were: MS/MS Ion Search with instrument type ESI-TRAP, Precursor tolerance 4 ppm and product ion tolerances ± 0.4 Da; Acrylamide (C), oxidation (M) and Carbamidomethyl (C) specified as variable modifications, enzyme specificity was trypsin, 1 missed cleavage was possible. Mascot search results confirmed that the purified protein in this study is the hexameric SH of *R. eutropha* H16 (Table 1)

### Step 3: soluble hydrogenase activity in solution and immobilized form

An uptake hydrogenase activity (H_2_ oxidation) assay was developed based on previously described methods [2], [7], [10]. Further electrochemical studies were performed using cyclic voltammetry (CV) with the enzyme immobilized on modified EPG (edge plane pyrolytic graphite) electrodes. Differently modified electrodes with the SH protein film applied were employed to study H_2_ oxidizing activity. The preliminary results demonstrated that the enzyme exhibits H_2_ oxidation activity at the surface of electrode and therefore this enzyme is a promising candidate for biochemical oxidation in hydrogen biofuel cell research.

### Materials

• Cary 100 UV–vis Spectrophotometer with Temperature Controller (Varian, Australia) at 30 °C
• Septum-sealed special optical glass cuvettes (Starna, Australia)
• 50 mM H_2_-saturated Tris/HCl buffer (pH 8.0)
• NAD+ solution (1 mM final concentration)
• Argon and H_2_ gas cylinders
• EPG electrodes
• Polymyxin for the electrode modification
• A platinum wire and Ag/AgCl as a control and a reference electrode, respectively
• MPG-2 potentiostat supplied with EC-Lab^®^ software (Bio-Logic, USA)

### Procedure

**Fig. 3:**
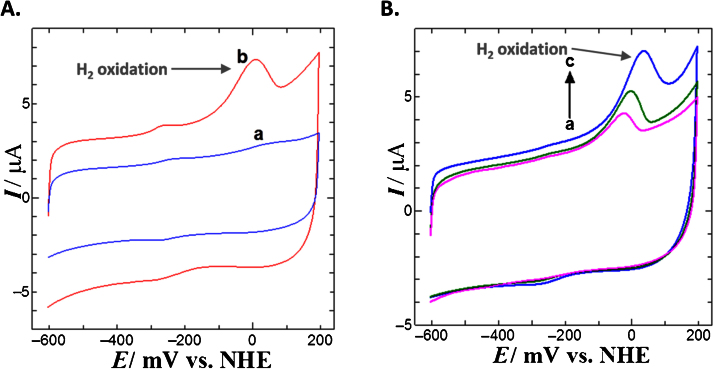
Cyclic voltammograms for pure SH immobilized on an EPG electrode. A) H_2_ oxidation in the presence and absence of Polymyxin with SH. Both electrodes were activated by NADH. 5 μL of activated SH only (a in blue) and mixture of 3 μL of activated hydrogenase and 2 μL of 2% of Polymyxin (b in red) are shown. **B)** H_2_ oxidation following different incubation times at an applied potential of −550 mV (vs. NHE). 5 μL of SH was applied on an EPG electrode. 5 min (a in pink), 10 min (b in green) and 30 min (c in blue). All cyclic voltametric experiments were carried out in 50 mM KPi buffer (pH 7.0) at a scan rate 50 mV s^−1^_._*I* − Current, *E* − potential, NHE − Normal Hydrogen Electrode. (For interpretation of the references to colour in this figure legend, the reader is referred to the web version of this article.)

1. In-solution enzyme activity assay: The hydrogen-oxidizing activity of a SH preparation was measured anaerobically with NAD^+^ as an electron acceptor on a Cary 100 UV–vis Spectrophotometer with Temperature Controller at 30 °C (NAD^+^). The SH solution, 100 μL, was added to 2.9 mL reaction mixture containing 50 mM H_2_-saturated Tris/HCl buffer (pH 8.0) and 1 mM NAD^+^ after 10 min Argon and 10 min H_2_ sparging to a septum-sealed special optical glass cuvette. For all gas sparging (both argon and H_2_) a pressure of 20 psi and flowrate of 4–5 mL/min was applied. The mixture was then pre-incubated at 30 °C (NAD^+^) for 5 min. NADH formation (ε = 6.22 mM^−1^ cm^−1^) was monitored with continual incubation for 10 min at 0.5 min intervals at 340 nm using the Cary WinUV software. The unit of enzyme activity is defined as the reduction of 1 μmol NAD^+^ per min. The absorbance change at 340 nm per minute (δA_340nm_/min) is calculated using the data obtained from the slope of a linear absorbance versus time plot.
2. Immobilized enzyme activity assay: Electrochemical analyses of the SH preparations purified in the present study were carried out by the method of CV [11]. The H_2_ oxidation activity of the enzyme preparations were investigated by forming a protein film on modified EPG electrodes. Polymyxin was used to immobilize the SH on an EPG electrode. In all electrochemical experiments, 50 mM KPi buffer at pH 7.0 was used. A platinum wire and Ag/AgCl were employed as a control and a reference electrode, respectively, and potentials were normalised vs. NHE (Normal Hydrogen Electrode). Potentials were controlled using the MPG-2 potentiostat with the scan rate of 50 mV s^−1^. Fig. 3 shows cyclic voltammograms recorded in H_2_-equilibrated 50 mM KPi buffer (pH 7.0) for a film of the SH immobilized on EPG electrodes. H_2_ oxidation activity was examined on the NADH activated electrodes in the presence and absence of polymyxin. 5 μL of activated hydrogenase did not show any activity in the absence of polymyxin, however, it displayed H_2_ oxidizing activity in the presence of polymyxin when an external potential was applied (Fig. 3A). The dependence of H_2_ oxidation activity of the SH (5 μL) on incubation time of the reaction at the polymyxin-coated electrode surface was also analyzed by applying a potential of −550 mV (vs. NHE). The hydrogenase activity was observed to increase as the incubation time was prolonged (Fig. 3B). These findings confirm that the enzyme preparation purified in this study is capable of oxidizing H_2_ when immobilized on this electrode.

## Figures and Tables

**Table 1:** Mass spectrometry results of SH subunits. Bands are labelled as 1–5 according to their vertical position on gels in a descending order as shown in Fig. 2. MOWSE scores are derived from ion scores as a non-probabilistic basis for ranking protein hits. The Exponentially Modified Protein Abundance Index (emPAI) offers relative quantitation of the proteins in a mixture based on protein coverage by the peptide matches in a database search result. Data represented here were obtained from the Mascot database server (Matrix Science).

Bands analyzed	Proteins detected	Database accession number	MOWSE score	Number of unique peptide identified	Sequence coverage, %	emPAI value
1	**HoxF** NAD-reducing hydrogenase diaphorase moiety large subunit [*R. eutropha* H16]	gi|38637753	4562	142	81	61.43
2	**HoxH** NAD-reducing hydrogenase moiety large subunit [*R. eutropha* H16]	gi|38637756	3401	112	71	48.36
3	**HoxU** NAD-reducing hydrogenase diaphorase moiety small subunit [*R. eutropha* H16]	gi|38637754	1692	61	87	194.61
4	**HoxY** NAD-reducing hydrogenase moiety small subunit [*R. eutropha* H16]	gi|32527093	474	18	61	7.91
5	**HoxI** [*R. eutropha* H16]	gi|38637758	2876	96	73	346.06
